# Diagnostic Performances of an Occupational Burnout Detection Method Designed for Healthcare Professionals

**DOI:** 10.3390/ijerph182312300

**Published:** 2021-11-23

**Authors:** Agathe Nguyen Huynh, Christine Besse, Zakia Mediouni, Emna El May, Yara Shoman, Isabelle Hansez, Irina Guseva Canu

**Affiliations:** 1 Center for Primary Care and Public Health (Unisanté), Department of Occupational and Environmental Health, University of Lausanne, 1066 Lausanne, Switzerland; agathe.nguyen.huynh@gmail.com (A.N.H.); zakia.mediouni@unisante.ch (Z.M.); e.elmay@gmail.com (E.E.M.); irina.guseva-canu@unisante.ch (I.G.C.); 2 Medical Direction of the Department of Psychiatry, CHUV, Les Cèdres (Cery), 1008 Prilly, Switzerland; Christine.Besse@chuv.ch; 3 Faculty of Psychology and Educational Sciences, University of Geneva, 1205 Geneva, Switzerland; 4 Unit of Promotion of Human Resources, Faculty of Psychology, Speech Therapy and Educational Sciences, University of Liège, 4000 Liège, Belgium; ihansez@uliege.be

**Keywords:** burnout, criterion validity, diagnostic tool, inter-rater reliability, patient-reported outcome measures (PROMS), Oldenburg Burnout Inventory (OLBI)

## Abstract

Background: We aimed to assess the validity (criterion and cross-cultural validity) and reliability of the first occupational burnout (OB) detection tool designed for healthcare professionals in Belgium in the context of Swiss medical practice. Methods: First, we assessed the sensitivity and specificity of the Tool. We developed this tool based on the consultation reports of 42 patients and compared its detection to the results of the Oldenburg Burnout Inventory (OLBI), filled-in by patients before a consultation. Second, we performed an inter-rater reliability (IRR) assessment on the OB symptoms and detection reached by the Tool between a psychiatrist, two psychologists, and an occupational physician. Results: The Tool correctly identified over 80% of patients with OB, regardless of the cutoff value used for OLBI scores, reflecting its high sensitivity. Conversely, its specificity strongly varied depending on the OLBI cutoff. There was a slight to fair overall agreement between the four raters on the detection of OB and the number of OB symptoms. Around 41% of symptoms showed a substantial to an almost perfect agreement, and 36% showed a slight to a moderate agreement. Conclusions: The Tool seems useful for identifying OB of moderate and strong severity in both the Belgian and Swiss contexts.

## 1. Introduction

There are no diagnostic standards for occupational burnout (OB), and none of the currently existing measurement tools could be used as a gold standard or comparator [1,2]. OB detection requires validated measurement tools on which healthcare practitioners and researchers can rely. The measurement tools that are currently available [3,4,5,6,7] are all patient-reported outcome measures (PROMS)—i.e., they rely solely on self-report—and their clinical validity has been questioned [8]. Indeed, PROMs should show vigorous psychometric properties to be utilized validly and reliably in medical research and practice [9]. In a recent systematic review on the validity of five PROMs, only the Copenhagen Burnout Inventory (CBI) and, to a lesser extent, the Oldenburg Burnout Inventory (OLBI) seemed to fulfil this requirement [10]. The insufficient validation of the first burnout PROMs has led to their multiplication and a marked heterogeneity in OB detection [2].

To address these issues, the Belgian Public Service for Employment, Work and Social dialogue (BPSEWS) developed a tool designed for healthcare professionals to use in clinical practice [11]. It aims to guide healthcare professionals through their consultation to establish the presence/absence and the frequency of typical OB symptoms and their relationship to work. The Tool was created in 2010 on the basis of a literature review and focus groups including 32 professionals from occupational medicine, insurance medicine, psychiatry, and psychology [12]. The Tool was tested by 346 general and occupational physicians during three months in 135,131 consultations. This confirmed that the list of symptoms was relevant, and in 2019, the Tool was then updated following nine individual interviews [13]. Its validity and reliability were investigated using the OLBI as a comparator. The Tool was also made available to healthcare professionals in the context of a national program aiming at the early detection of OB in workers exposed to psychosocial risk factors, their treatment, and subsequent two-year follow-up for secondary and tertiary prevention purposes [14].

The conceptualization of occupational burnout has always been problematic. Schaufeli and Taris [15] suggested that burnout should be considered as a work-related phenomenon consisting of at least two dimensions that can be measured generally or specifically. The OLBI conceptualizes occupational burnout as two dimensions: exhaustion and disengagement from work [4]. The exhaustion dimension of the OLBI covers affective, physical, and cognitive aspects, whereas the disengagement dimension covers the relationship between the worker and his/her job (regarding engagement and identification). In Belgium, Hansez et al. consider burnout as a temporal process consisting of four stages of burnout, allowing an underlining of the complexity of its diagnosis [16]. The Tool is the first to assess the clinical judgement of a burnout diagnosis with the help of a structured consultation and detection of the symptoms and patterns of occupational burnout [17].

The importance of PROMs is widely accepted in clinical practice and research [18]. Nevertheless, they are often not enough for diagnostic purposes [19], and the use of hetero-assessment tools is still necessary. In a recent survey of Swiss physicians in primary and occupational healthcare, 88% said that they would find it interesting to integrate an OB diagnostic tool into their practice [20]. Therefore, we aimed at testing the Tool in a Swiss context to establish whether it could be implemented in Swiss medical practice. Our objective was to assess the criterion validity, reliability, and cross-cultural validity of the Tool, when comparing it with OLBI.

## 2. Materials and Methods

### 2.1. Study Sample

We used a convenience sample of patients received at the Unisanté “Work and Suffering” Consultation (WSC) between 2010 and 2013. The WSC is a multidisciplinary consultation, specialized in helping people in situations of OB, conflict, stress, violence, or harassment at work [21]. Between 2010 and 2013, the WSC operated in its pilot form, and all patients were asked to complete several PROMs just before the consultation, including the OLBI [4]. When the consultation entered into its regular operation, its duration was shortened, and the use of PROMs was abandoned. We included WSC patients for whom a completed OLBI was available in their medical record along with the WSC detailed report.

### 2.2. Study Design

We implemented a diagnostic research study design [22]. We assessed the validity (i.e., criterion and cross-cultural validity) and reproducibility (i.e., inter-rater reliability) of the Tool with the use of another measurement tool (i.e., the comparator). We chose the OLBI (the OLBI measures two dimensions of OB: exhaustion and disengagement and it includes 16 items, on which patients have to rate their agreement on a scale from one to four) as a comparator because it was used in the pilot phase of the WSC, and it was available in medical records, but also because the OLBI is the second-most used PROM of OB after the MBI and the second-most validated one after the CBI [10]. Moreover, Hansez et al. (2019) also used the OLBI as a comparator, allowing further comparison of the results [13].

### 2.3. A New Method for OB Detection by Healthcare Professionals

We used the latest version of the Tool, which was amended to conform to the Swiss medical vocabulary (via the reformulation of the reasons for consultation and alternative diagnoses) and reduced to a more condensed format. This resulted in a two-page document, including questions about the type of medical examination; the reasons for consultation; the socio-demographic characteristics of the patient; a list of the physical, cognitive/affective, and behavioral symptoms typical of OB cases; the relationship to work (i.e., work-related risk factors and missing resources, the importance of work for the patient); the current or past sick leaves; and the detection of OB (including a nota bene indicating that, in order to be diagnosed with OB, the patient needs to report at least two, six, and two physical, cognitive/affective and behavioral symptoms, respectively, and that his/her complaints need to originate primarily from work). These sections all contained multiple choices, allowing the healthcare professional to tick the boxes corresponding to the situation of the patient (See Supplementary Material).

We used the WSC reports instead of real consultations with patients. The WSC reports were written and signed by an occupational physician (usually assisted by a resident in occupational medicine) and co-signed by a psychiatrist who further assessed the presence of psychiatric disorders. The WSC reports were approximately 8 pages long and contained a description of the past and current occupations of the patient (tasks, responsibilities) and the patient’s perception of them, an evaluation of the impact of work factors on the physical and mental health of the patient, the personal history of the patient (family relationships, significant life events), the medical and psychiatric anamnesis of the patient (medical history, health habits), and a summary of the multidisciplinary discussion concerning the patient and his/her treatment.

We distributed a set of the anonymized WSC reports, without the OLBI results, to four raters with different backgrounds and experience: one psychiatrist, one occupational physician, one psychologist, and one master’s student in psychology. Each rater was asked to fill in one Tool on the basis of each WSC report exclusively. The raters were instructed to complete the Tools as exhaustively as possible. For example, if a symptom was not clearly stated in the report but the rater could read its presence between the lines, the symptom was reported. After having fully completed the Tool, the raters were instructed to refer to the cutoff values in the nota bene for their final detection of OB. To ensure the anonymity of the raters in the analyses, a random number was distributed to each of them by the study coordinator.

### 2.4. Statistical Analysis

#### 2.4.1. Validity of the Tested Method

To assess the validity of the Tool, we tested the criterion and cross-cultural validity. For the criterion validity we used the OLBI as a comparator. We calculated the Tool’s sensitivity, positive predictive value (i.e., the probability that patients who are identified with OB by the Tool truly have OB) and negative predictive value [23]. The OLBI does not identify OB in a dichotomous manner but results in a global score between 16 and 64. Therefore, we considered two possible cutoff values in our analyses: the first cutoff value was 30, corresponding to mild and high severity burnout, while the second cutoff value was 44, corresponding to only high severity burnout. These cutoff values were established by Hansez et al. (2019) based on the distribution of OLBI scores in a large representative sample of Belgian workers [13]. We first conducted the validity analyses considering that patients with an OLBI score above 30 suffered from OB and then considering that patients with an OLBI score above 44 suffered from OB. Finally, we interpreted the diagnostic usefulness of the method (if the sum of sensitivity and specificity was at least 1.5). According to Power et al. [24], the method is considered useful, halfway between 1, which is useless, and 2, which is perfect. The analyses were performed using Stata 16 (StataCorp LLC., College Station, TX, USA) [25].

#### 2.4.2. Reliability of the Tested Method

We performed inter-rater reliability (IRR) analyses to assess the degree of agreement between four raters regarding OB detection and the identification of each of the 22 symptoms of the Tool. Our design being fully crossed, we first calculated Cohen’s kappa [26] for all rater pairs and then used the arithmetic mean of these estimates to provide an overall index of agreement [27]. Following Hallgren’s (2012) [28] recommendations, we investigated the marginal distribution of each variable to apply the appropriate correction to Cohen’s kappa. When a prevalence problem was identified (the marginal distributions of observed ratings fell into one category of ratings at a much higher rate over another), the correction was performed according to Byrt, Bishop, and Carlin [29], resulting in Byrt’s kappa. When a bias problem was identified (the marginal distributions of specific ratings were substantially different between coders), Siegel and Castellan’s correction of Cohen’s kappa was applied [30]. All values of kappa were interpreted according to Landis and Koch [31] (values between 0.0 and 0.2 corresponded to a slight agreement, between 0.21 and 0.40 to a fair agreement, between 0.41 and 0.60 to a moderate agreement, between 0.61 and 0.80 to a substantial agreement, and between 0.81 and 1.00 to an almost perfect/perfect agreement).

Last, in order to assess the degree of agreement in the total number of symptoms identified for each patient (continuous variable), IRR was assessed using a two-way mixed, absolute, single-measure inter-class correlation (ICC) [32]. The value of the ICC was interpreted according to Cicchetti (when the reliability of the coefficient was below 0.40, the level of significance was considered poor, when it was between 0.40 and 0.59, the level of clinical significance was fair; when it was between 0.60 and 0.74, the level of clinical significance was good; and when it was between 0.75 and 1.00, the level of clinical significance was excellent.) (1994) [33]. All IRR analyses were run on R (R Foundation for Statistical Computing. Vienna, Austria) [34] following Hallgren’s (2012) procedure [28].

## 3. Results

### 3.1. Characteristics of the Patient Sample

Overall, 65 patients were received at the WSC between 2010 and 2013, and 42 of them completed the OLBI and were included in the study. They were mostly women (74%) and had a median age of 46 (interquartile range (IQR) = 20). A total of 67% were in a relationship, and 33% were single. A total of 69& worked full-time, and 26% worked part-time, while 5% were not employed. Out of the 40 workers, most were employees (72%), 25% were managers, and 3% did not state their status. Most worked in the private sector (64%), and 5% worked in the public sector, while 17% did not provide this information. The patients’ median seniority in the company was six years (IQR = 7.5). More than half of the workers had a permanent contract (67%), 5% had a fixed term contract, 20% were dismissed or in the process of being dismissed, 5% had quit or were in the process of quitting, and 3% did not state the type of their contract. Around 55% of patients were on sick leave at the time of the consultation because of the symptoms they reported during the consultation, and 64% had been on sick leave in the last twelve months for the same reasons.

### 3.2. Validity of the Tool

Table 1 summarizes the number of patients diagnosed with OB according to the comparator (the OLBI) and the tested method (the Tool). The sensitivity of the Tool was above 0.80 regardless of the OLBI cutoff value considered (Table 2). Conversely, for the specificity assessment, the choice of cutoff value seemed determinant. When the cutoff > 30 was used, the specificity of the Tool was perfect, although there was only one true positive patient. However, when a more stringent cutoff value was applied (>44), the specificity largely dropped to 25%. The sum of sensitivity and specificity was 1.83 when a cutoff > 30 was used, meaning that the test could be considered useful according to Power et al. (2013) [24]. However, this was not the case when a cutoff > 44 was used, i.e., the sum of sensitivity and specificity was 1.17.

### 3.3. Reliability of the Tool

The raters reported an average of 12 symptoms in total per Tool (SD = 0.23), including three physical symptoms (SD = 0.07), seven cognitive and affective symptoms (SD = 0.17), and two behavioral symptoms (SD = 0.07). They reported on average five work-related risk factors per Tool (SD = 0.15) and five resources that the patient was lacking at work (SD = 0.15). On average, the four raters diagnosed OB in 53% of cases.

The IRR analyses suggested that raters had a slight to a fair agreement in OB detection when using the Tool (Table 3). Similarly, the ICC on the total number of symptoms reported by the raters was in the fair range of clinical significance, ICC = 0.50. When looking at the 22 individual symptoms, 9 had Cohen’s and corrected kappas in the substantial to an almost perfect range (41%), and 8 had Cohen’s and corrected kappas in the slight to moderate range (36%).

## 4. Discussion

### 4.1. Summary of the Results

The criterion validity analyses with the OLBI as a comparator revealed that the Tool correctly identified over 80% of patients with OB, regardless of the cutoff value used for OLBI scores, reflecting its high sensitivity. Conversely, its specificity strongly varied depending on the OLBI cutoff, the latter still being undetermined. The IRR varied significantly from symptom to symptom: around 40% of symptoms showed a substantial to almost perfect agreement, while slightly more than one third showed a slight to moderate agreement. There was a slight to fair overall agreement between the four raters on the detection of OB and the number of associated symptoms.

### 4.2. Cross-Cultural Validity of the Tool

Comparing our results to those of Hansez et al. (2019) [13], who investigated the diagnostic accuracy of the Tool with OLBI as a comparator in Belgium, we had slightly higher sensitivity values than theirs (cutoff > 30, sensitivity = 66%; cutoff > 44, sensitivity = 77%). Interestingly, they also found a perfect specificity when using the cutoff > 30, and it dropped to 55% when using the cutoff > 44. Moreover, we both found that the sum of the sensitivity and specificity exceeded the critical value of 1.5, above which a test can be considered useful, when using the cutoff > 30 but not when using the cutoff > 44 [24]. From these results, Hansez et al. concluded that the Tool could be used in combination with a PROM, and they made it available in their national program with the OLBI. We consider that, in the Swiss context, the use of one validated tool would be more realistic and useful. The Tool is the only non-PROM tool available. After some amendments to increase its specificity and the confirmation of its cross-cultural validity, assessed using a true diagnostic standard (still nonexistent) or a precisely determined cutoff for the OLBI (ongoing research), we could firmly decide whether we should recommend its use.

The results suggest that burnout can be considered as a temporal process consisting of stages even in Swiss context. It was mentioned in the literature that burnout follows certain stages (from exhaustion to reduced professional efficacy) [35,36]. Nevertheless, the Tool is based on the theory that burnout consists of four stages: stage zero being a symptom-free stage, called “Engagement in work with idealistic enthusiasm”. The three further stages of burnout development are “Weakening of the idealism”, “Emergence of protective withdraw”, and “Burnout” [16]. A better understanding and investigation of these stages will allow the enhanced prevention, diagnosis, and treatment of burnout.

### 4.3. Reliability of the Tool

The results suggesting a slight to fair reliability of the medical detection based on the Tool between four raters deserve careful interpretation. It is interesting to note that the two most experienced raters often did not agree with the detection of OB reached by the Tool (for 29% and 57% of patients, respectively). In the large majority of these cases, the raters recognized that there was a process of OB but that this process had either been exceeded, i.e., the symptoms now reached the threshold for a psychiatric disorder (Code F ICD-10 Diagnosis) [37], or that a pre-existing psychiatric disorder might have contributed to the process of OB and was the predominant feature of the crisis’s symptomatology. The frequent occurrence of psychiatric comorbidities in our sample can be explained by the presence of relatively severe and longstanding cases. Indeed, more than half of patients were on sick leave at the time of the consultation, and 64% had been on sick leave in the prior twelve months. This makes it challenging for a standardized tool to capture the richness and complexity of the specialized multidisciplinary medical expertise contained in the WSC reports. These situations are however less common in other, less specialized consultations, such as those in general medical practice. Recommendations on the Tool’s use in the presence of comorbidities could be helpful and should improve the inter-rater reliability.

### 4.4. Study Limitations

The OLBI, like any other currently available PROM, cannot be considered as a gold standard for identifying OB. We considered the OLBI as an acceptable comparator, as it was shown to have better content and construct validity as well as internal consistency than other most-used PROMs [10]. Moreover, in accordance with a harmonized definition of OB [1], it measures the exhaustion dimension. Its use allowed us to compare our results with those of Hansez et al., using the same cutoff values. The latter was established based on the distribution of OLBI scores in Belgian workers [13] as a first step towards cutoff definition, but there is still no cutoff value allowing dichotomization of OLBI scores. Its forthcoming establishment should enable a true assessment of the Tool’s validity, both in Belgium and in Switzerland.

For unknown reasons, 19 of the 65 patients who consulted within this period (29.2%) did not complete the OLBI. A comparison of their sociodemographic data, dates, and motives for WSC with those of patients who did complete the OLBI, even incompletely (N = 4), did not reveal any significant difference (results not shown). This allows us to rule out a potential selection bias that might have occurred through the exclusion of patients with no or an incomplete OLBI.

A limitation of this study is that the raters completed the Tool on the basis of consultation reports and not real face-to-face consultations. This is not the way the Tool is meant to be used. However, the reports followed a standardized format and contained detailed and validated information on the patient. Moreover, this method had the advantage of presenting exactly the same material to all raters. This allowed us to truly test the robustness of the Tool. If, alternatively, we had asked the raters to use the Tool four times on the same patient, there could have been variations in the content of the consultation due to the repetitive questioning of the patient, the context of the consultation, or differences in the physician–patient interaction. Moreover, this would have been logistically difficult and would have prolonged the WSC.

Another limitation of this study is that the data collected from patients could not be updated to capture any significant changes in the sample, especially after the pandemic of COVID-19. Unfortunately, in the current form of “Work and Suffering” consultations, patients do not complete PROMs, and only anamnesis and clinical examination reports are available. Additionally, it is necessary to assess the validity and reliability of the Tool when comparing it against additional comparators with a precise and validated cutoff value—for instance, such as the CBI or Burnout Assessment Tool (BAT) [38] in future research.

## 5. Conclusions

The need for validated OB measurement tools has been stressed by both researchers and healthcare practitioners [2,20,39,40]. This study assessed the performance of the first OB measurement tool to rely on the report of healthcare professionals and not patients. According to our results, the Tool is promising. However, its detection of OB using a better comparator with a precise and validated cutoff value is necessary. The potential lack of specificity and inter-rater reliability could be rectified through an appropriate amendment of the Tool. The adapted version of the Tool could be further tested in practice, during face-to-face consultations, particularly in medical settings, which are not specialized in occupational health.

## Figures and Tables

**Table 1:** Number of patients diagnosed with occupational burnout according to the reference and the tested methods.

Method	Detection+	Detection−
Reference method (cutoff > 30, occupational burnout of mild and high severity)	41 (98%)	1 (2%)
Reference method (cutoff > 44, occupational burnout of high severity only)	25 (60%)	17 (40%)
Tested method (binary medical detection of occupational burnout)	34 (81%)	8 (19%)

**Table 2:** Validity of the Tool.

Cutoff for OLBI ^1^ Score	Sensitivity	Specificity	PPV ^2^	NPV ^3^
>30, occupational burnout of mild and high severity	0.83	1	1	0.13
>44, occupational burnout of high severity only	0.88	0.29	0.65	0.63

^1^ Oldenburg Burnout Inventory, ^2^ positive predictive value, ^3^ negative predictive value.

**Table 3:** Inter-rater reliability of the symptoms identified and the detection reached by the four raters using the Tool.

Variable	Cohen’s Kappa	Problem(s)	Corrected Kappa
Sleep problems	κ_Co_ = 0.71 ****	Prevalence	κ_B_ = 0.90 *****
Lower energy	κ_Co_ = 0.31 **	Prevalence	κ_B_ = 0.18 *
Neuro-vegetative/functional complaints	κ_Co_ = 0.69 ****	Prevalence	κ_B_ = 0.62 ****
Profound fatigue	κ_Co_ = 0.66 ****	Prevalence	κ_B_ = 0.72 ****
Lower motivation at work	κ_Co_ = 0.56 ***	Prevalence	κ_B_ = 0.26 **
		Bias	κ_S&C_ = 0.19 *
Frustration	κ_Co_ = 0.55 ***	Bias	κ_S&C_ = 0.21 **
Irritability	κ_Co_ = 0.73 ****	Prevalence	κ_B_ = 0.83 *****
Depressive mood	κ_Co_ = 0.49 ***	Prevalence	κ_B_ = 0.49 ***
		Bias	κ_S&C_ = 0.35 **
Rumination	κ_Co_ = 0.63 ****	Prevalence	κ_B_ = 0.83 *****
Ambivalence: leave job or stay	κ_Co_ = 0.55 ***	Prevalence	κ_B_ = 0.21 **
		Bias	κ_S&C_ = 0.13 *
Anxiety	κ_Co_ = 0.41 ***	Prevalence	κ_B_ = 0.66 ****
Lower self-esteem	κ_Co_ = 0.78 ****	-	-
Lower focus	κ_Co_ = 0.86 *****	-	-
Lower feeling of competence	κ_Co_ = 0.68 ****	Prevalence	κ_B_ = 0.47 ***
		Bias	κ_S&C_ = 0.44 ***
Lower feeling of control	κ_Co_ = 0.54 ***	Prevalence	κ_B_ = 0.18 *
		Bias	κ_S&C_ = 0.03 *
Lower memory	κ_Co_ = 0.95 *****	Prevalence	κ_B_ = 0.90 *****
Lower idealism	κ_Co_ = 0.72 ****	Prevalence	κ_B_ = 0.45 ***
		Bias	κ_S&C_ = 0.06 *
Change in attitude towards others	κ_Co_ = 0.61 ****	Prevalence	κ_B_ = 0.27 **
		Bias	κ_S&C_ = −0.05
Tendency to isolate oneself	κ_Co_ = 0.79 ****	-	-
Absenteeism for illness	κ_Co_ = 0.50 ***	Prevalence	κ_B_ = 0.43 ***
		Bias	κ_S&C_ = 0.44 ***
Lower performance	κ_Co_ = 0.73 ****	Prevalence	κ_B_ = 0.49 ***
		Bias	κ_S&C_ = 0.35 **
Aggressiveness, impulsivity	κ_Co_ = 0.70 ****	Bias	κ_S&C_ = 0.49 ***
Detection of occupational burnout	κ_Co_ = 0.38 **	Prevalence	κ_B_ = 0.21 **
		Bias	κ_S&C_ = 0.10 *

* slight agreement, ** fair agreement; *** moderate agreement, **** substantial agreement; ***** almost perfect/perfect agreement.

## Data Availability

In accordance with ethics committee’s permission, the raw medical and diagnostic data cannot be provided. All other material used in this study is available as Supplementary Material.

## Supplementary Materials

The following are available online at https://www.mdpi.com/article/10.3390/ijerph182312300/s1, File S1: Tool for the early detection of occupational burnout.
