# Supplementary figures and images for: Diagnostic Performances of an Occupational Burnout Detection Method Designed for Healthcare Professionals
Source: Int J Environ Res Public Health. 2021 Nov 23;18(23):12300. doi: 10.3390/ijerph182312300 (PMC8657176; doi:10.3390/ijerph182312300)

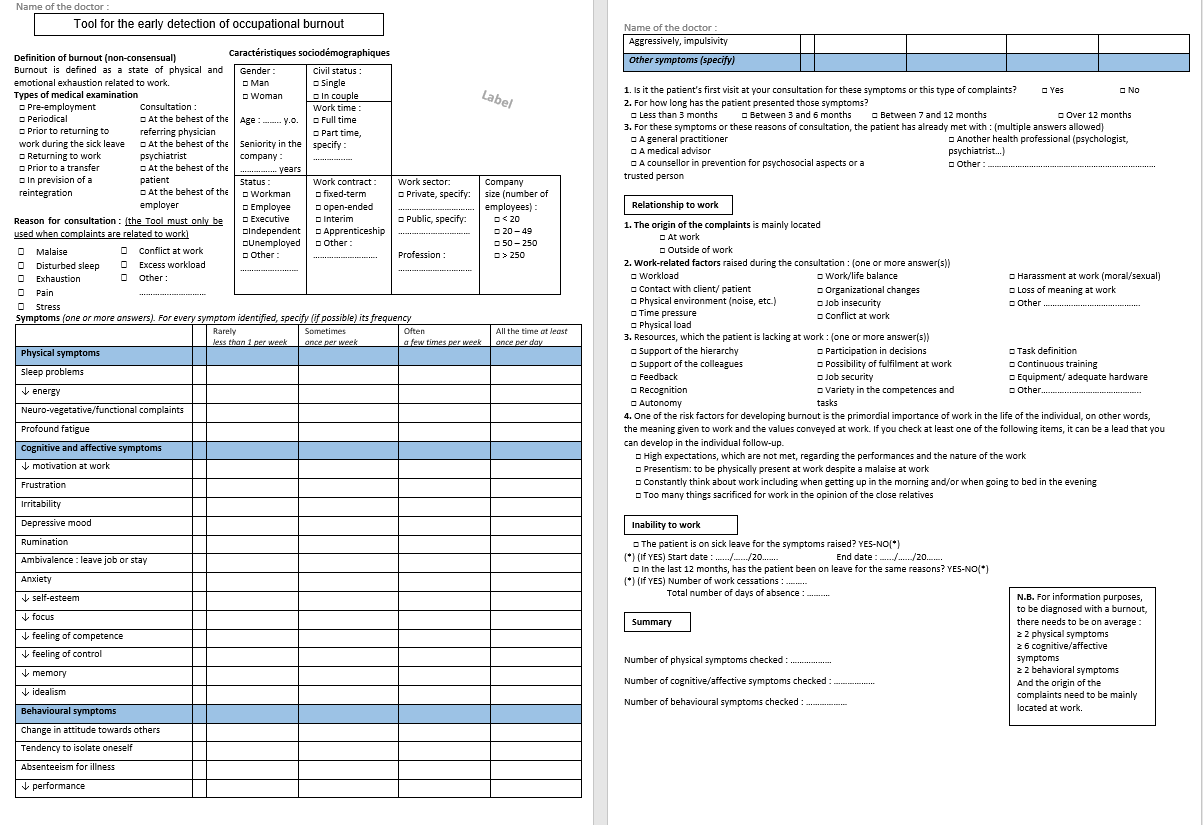

Supplement: Supplementary file 1 [file ijerph-18-12300-s001.zip › ijerph-1428819.png]
